# Two Sides of the Same Coin: Environmental and Health Concern Pathways Toward Meat Consumption

**DOI:** 10.3389/fpsyg.2020.578582

**Published:** 2020-12-16

**Authors:** Amanda Elizabeth Lai, Francesca Ausilia Tirotto, Stefano Pagliaro, Ferdinando Fornara

**Affiliations:** ^1^Department of Psychology, Norwegian University of Science and Technology, Trondheim, Norway; ^2^Department of Psychology, University of Plymouth, Plymouth, United Kingdom; ^3^Department of Education, Psychology, Philosophy, University of Cagliari, Chieti, Italy; ^4^Group Processes and Morality Lab (GPM-Lab), Department of Neuroscience, Imaging and Clinical Sciences, University of Studies G. d'Annunzio Chieti and Pescara, Cagliari, Italy

**Keywords:** value belief norm theory, health concern, biospheric values, personal norm, social norms, simulated meat purchase

## Abstract

The dramatic increase of meat production in the last decades has proven to be one of the most impacting causes of negative environmental outcomes (e.g., increase of greenhouse emissions, pollution of land and water, and biodiversity loss). In two studies, we aimed to verify the role of key socio-psychological dimensions on meat intake. Study 1 (*N* = 198) tested the predictive power of an extended version of the Value-Belief-Norm (VBN) model on individual food choices in an online supermarket simulation. In an online survey, participants were directed to a virtual shop and asked to buy food within a set amount of money. Subsequently, they completed measures of behavioral intention, the VBN constructs (values, general pro-environmental beliefs, awareness of consequences, ascription of responsibility, and personal norm), and social norms (injunctive and descriptive). The outcome variable was operationalized in terms of percentage of expenses dedicated to meat and processed meat items, which provided a more robust behavioral measure than the common self-reported ones. Results confirmed the VBN sequential path, showing direct effects of biospheric values and descriptive norm on personal norm. Furthermore, a proof of validity for the new behavioral measure was provided (medium-sized correlation with behavioral intention). Study 2 (*N* = 218) aimed at verifying whether the meat consumption could be also motivated by a health concern, reflecting individual (cost/benefit) considerations, besides pro-environmental drivers. Results showed the direct impact of health concern and confirmed the indirect role of biospheric values and descriptive norm (*via* personal norm) on meat intake. This evidence would suggest the use of multiple-frame messages, highlighting both pro-environmental and health consequences, for meat consumption reduction. Nevertheless, the different implications of moral (e.g., environmental concern) vs. non-moral motivators (e.g., health concern) for reducing meat intake need to be stressed: indeed, the first drivers are more central for self-identity and for engaging in environmental citizenship behaviors.

## Introduction

The investigation of socio-psychological factors influencing people’s willingness to reduce meat consumption has become a critical research line in climate change studies. Indeed, reducing meat intake is a key mitigation response to environmental issues (Poore and Nemecek, 2018; Arneth et al., 2019), being recognized among the highest impact actions to reduce green gas emissions (Wynes and Nicholas, 2017; Dubois et al., 2019). Benefits from a low-meat diet have been also demonstrated for the reduction of human health risks, related to type II diabetes, cancer, coronary heart disease, and mortality (Abete et al., 2014; Clark et al., 2019). Therefore, meat consumption has consequences for both sustainability and health-related outcomes (González et al., 2020).

About 97% of European adults are currently identified as meat consumers (Cocking et al., 2020). Looking at the future scenario, people in European countries are predicted to reduce the intake of pork and beef, while increasing the consumption of poultry and sheep meat; this will only lead to a slight drop of meat consumption by 2030 (from 69.3 to 68.7 kg per capita; EC, 2018). In order to further reduce the per capita consumption of meat in European countries, socio-psychological factors that could potentially drive and inform campaigns aimed at raising people’s awareness toward the impact of this behavior need to be investigated. In this regard, simply providing information about consequences of a certain behavior does not always translate into a change in behavior, but it is rather a precondition of it (e.g., Schultz, 2002; Kahan et al., 2012; Geiger et al., 2019). For example, Heeren et al. (2016) showed that the knowledge of the impact of pro-environmental behaviors has no effect on behavior when controlling for factors such as attitudes, norms, and perceived behavioral control. Furthermore, established habits may hinder the intention to consume less meat (Klöckner and Blöbaum, 2010; Cheah et al., 2020). Behaviors are embedded within a social context, which gives people direction through different levels and degrees of internalized values (e.g., Schwartz and Howard, 1984), norms (Thøgersen, 2006), and motivations (e.g., Ryan and Deci, 2000). Such dimensions should be thus considered when delivering information aimed at increasing people’s knowledge of the consequences of a meat-based diet, with the final purpose of triggering a change in meat consumption.

We aimed to contribute to the literature on meat consumption reduction by investigating relevant socio-psychological aspects that are associated with pro-environmental behaviors. In order to do that, we tested an extended version of the Value-Belief-Norm (VBN) theory (Stern, 2000) that includes social norms in explaining meat purchasing behavior (Study 1), and then we evaluated the additional effect of an egoistic-utilitarian pattern represented by health concern on the same outcome variable (Study 2). In addition, an important purpose was to provide the first validation of a simulated behavior measure of meat consumption by asking the participants to purchase food on an online shopping platform. Such a purpose was motivated by the intention to overcome the well-known limitations of usual self-report questionnaire measures (Webb and Sheeran, 2006; Lange and Dewitte, 2019). In the next section, the theoretical framework will be briefly explained.

### Theoretical Background

#### Environmental and Health Concerns in Meat Consumption

According to literature, people are motivated to reduce their meat consumption for different reasons, e.g., animal welfare, environmental, and health concerns (see, for example, Sanchez-Sabate and Sabaté, 2019; for a systematic review on the field). These motivations are not mutually exclusive, however, it is possible to identify a trend, where animal-rights and ecological concerns are more likely to be found in those who completely exclude meat from their diet, whereas less morally relevant reasons, such as health concern, seem to mostly motivate those who deliberately choose to only reduce meat consumption (De Backer and Hudders, 2014, 2015; Rosenfeld et al., 2020). Among all the dietary inclinations, environmental concern is one of the less frequent reasons for reducing meat consumption. In other words, those genuinely motivated by ecological concern are still a small minority (Sanchez-Sabate and Sabaté, 2019). This could probably be due to the laypeople’s overall underestimation of the influence of meat production on the environment (de Boer et al., 2013; Macdiarmid et al., 2016; Lentz et al., 2018). In fact, when people believe that reducing meat consumption is beneficial for the environment, they are more likely to intend to quit eating meat (Truelove and Parks, 2012). This is in line with a more recent study in which environmental concern predicted the willingness to reduce meat consumption through the belief that reducing meat intake is an effective mitigation strategy for climate change (Ginn and Lickel, 2020).

Meat intake is also generally associated with both positive and negative health beliefs: in fact, some individuals may consider meat as an important source of energy and essential nutrients, such as high-value proteins (Godfray et al., 2018). At the same time, it is associated with the risk of developing chronic diseases (e.g., type II diabetes, cancer, coronary heart disease, and mortality; Abete et al., 2014; Clark et al., 2019), and it can also generate emotions of disgust when associated with animal diseases, such as bovine spongiform encephalopathy (BSE), commonly known as mad cow disease (Palomo-Vélez et al., 2018).

Research has shown that meat reducers, those who deliberately choose to limit the amount of meat consumed, are mostly motivated by health concerns and personal reasons (Mullee et al., 2017; Malek et al., 2019). Among pro-environmental behaviors, meat consumption reduction was, in fact, indicated as the least related to pro-environmental values, being driven especially by health concern (Jagers et al., 2017). The positive role of the health path in influencing meat consumption reduction is also supported by other studies that have tested the effect of message frames (Bertolotti et al., 2016, 2020) and the effect of a text message intervention (Carfora et al., 2019). On the other hand, as previously discussed, health concern has also been reported as one of the key reasons for eating meat regularly (Piazza et al., 2015; Neff et al., 2018; Stea and Pickering, 2019). These results showed a possible co-occurring valence associated with the healthiness of meat products.

Persuasive messages focusing on either environmental or health consequences of red (and processed) meat were found to promote positive attitudes toward its reduction and, in turn, to impact on the target behavior (Carfora et al., 2019). Similarly, Vainio et al. (2018) found that communicating health and sustainability benefits of eating less meat was positively associated with the intention to reduce meat consumption, but only among those with pre-existing strong negative beliefs regarding meat consumption (Vainio et al., 2018). However, Cheah et al. (2020) found different patterns of association. Perceived health benefits of reducing meat consumption were an important driver of the intention to reduce meat consumption, while environmental concern did not show the same significance.

#### Beliefs and Values in Meat Consumption

As mentioned earlier, previous studies show that environmental reasons are rarely mentioned as a motivation to reduce meat or to exclude meat from one’s diet. This may be due to the fact that the lack of information about environmental consequences of meat consumption undermines the development of individuals’ beliefs about the impacts of meat reduction on the environment (see, for example, Hartmann and Siegrist, 2017, for a systematic review on the topic). Beliefs about the positive effect of reducing meat consumption are based on the information that people hold and, more importantly, on their willingness, motivation, and ability to look for and process that information from different sources (e.g., scientific and commercial information sources; Vainio, 2019). The extent to which information about the environmental consequences of a behavior are sought and shaped into beliefs is influenced by the degree of environmental concern and is led by people’s values. As claimed by Stern et al., (1995, p. 726), “values and worldview act as filters for new information and ideas. Information congruent with an individual’s values and worldview will be more likely to influence beliefs and attitude.” Values have been conceptualized as “the criteria that people use to select and justify actions and to evaluate people (including the self) and events” (Schwartz, 1992, p. 1). Therefore, values act as guiding principles both in searching and evaluating the information on which one’s own beliefs are based.

Values can be depicted in two bipolar dimensions, i.e., self-enhancement (focus on the self) vs. self-transcendence (focus on the others) and openness to change vs. conservation (Schwartz, 1992). Making reference to the first dichotomy, Stern et al. (Stern et al., 1993; Stern and Dietz, 1994) developed a classification of values related to environmental issues and distinguished between egoistic (i.e., self-enhancement), altruistic (i.e., self-transcendence), and biospheric values (i.e., those values related to concern for nature and the environment). Generally, altruistic and biospheric values have emerged as positively associated with pro-environmental behaviors, whereas egoistic values have shown a negative relationship with such behaviors (Stern and Dietz, 1994; Stern et al. 1998; Corraliza and Berenguer, 2000). This pattern emerged also for meat consumption, since a self-enhancement value orientation was found related to a higher meat consumption compared to the self-transcendent one, whereas the latter predicts a lower meat intake (Graham and Abrahamse, 2017). To explain the nature of these relationships, Verma et al. (2019) argued that often the personal costs related to the pro-environmental actions overshadow the personal aids; therefore, actions motivated by egoistic values do not lead to behaving pro-environmentally. However, they also postulate that when the perceived benefits to self (e.g., good health and better quality of life) outweigh the personal costs, individuals then chose to behave in an eco-friendly way (Verma et al., 2019). Prakash et al. (2019) found, indeed, that both altruistic and egoistic values may lead to a positive impact on consumers’ attitude toward eco-friendly packaged goods. This is in line with Kareklas et al. (2014) findings, which show the positive effect of egoistic considerations on organic food purchase and by the work of Herziger et al. (2020) on the positive effect of egoistic appeals on consumption curtailment. Therefore, also utilitaristic reasons based on selfish motivations can trigger pro-environmental behaviors.

However, biospheric and altruistic values are shown to provide a more stable ground for pro-environmental behaviors than egoistic values (Schultz, 2001; De Groot and Steg, 2009). Behaviors operated under the influence of self-transcendent values (altruistic and biospheric) are morally relevant, therefore, even though there is not an apparent direct individual benefit in the short-term, such behaviors actually offer people a moral satisfaction in terms of a positive emotional reward named as “warm glow.” Warm glow is explained as “the feeling of well-being related to the contribution to a good cause” (Hartmann et al., 2017, p. 44). Self-transcendent values are also associated with self-determined motivations to act pro-environmentally (De Groot and Steg, 2010). When a behavior is self-determined, it involves a sense of voluntariness and reflects one’s interests or values (“I enjoy contributing to the environment”; Pelletier et al., 1998). More internalized/intrinsic motivations have the advantage to promote long-term pro-environmental behaviors (Osbaldiston and Sheldon, 2003). Therefore, important implications of the different drivers (i.e., biospheric vs. egoistic values) should be accounted for.

#### Value-Belief-Norm Theory and Social Norms

The influence of value systems on pro-environmental behaviors has been addressed by the VBN theory, which was formulated by Stern et al. (1999) for explaining public support for environmental movements. This theory is an extension of the Norm Activation Model (NAM: Schwartz, 1977) of altruism, proposing that people engage in helping behaviors if they are, first, aware of a situation of threat or danger; in other words, they should be aware of consequences of not coping with the problem. Secondly, people should ascribe the responsibility of these helping actions to themselves. If both the aforementioned psychological conditions are met, then feelings of moral obligation (i.e., the moral or personal norm) to help are activated and, in turn, they stimulate the requested helping behavior.

The extension operated by Stern et al. (1999) refers not only to other people in need of help, as it is postulated by NAM, but also other valued objects (e.g., the self, other species, and the biosphere) can be targeted, such as the self, other species, and the biosphere. Thus, people who especially value other species would be concerned about threatening environmental conditions. Hence, the activation of problem awareness depends on the possession of values and pro-environmental worldviews. In sum, the VBN theory proposes that pro-environmental action stems from a causal chain including values, general pro-environmental worldviews, awareness of consequences, ascription of responsibility, personal norm and, finally, the outcome behavior. This model has received empirical evidence for various kinds of pro-environmental behaviors, such as energy-related choices (Steg et al., 2005; Abrahamse and Steg, 2011; Fornara et al., 2016), urban travel choices (Lind et al., 2015; Ünal et al., 2019), climate change-related behaviors in farmers (Zhang et al., 2020), preservation of nature and biodiversity (Fornara et al., 2020), antinuclear behavioral intention (Prati and Zani, 2013), and residents’ behavior in touristic sites (Zhang et al., 2014). Nevertheless, there is a substantial lack of studies testing the VBN for food choices, and specifically for meat consumption, except for a study focusing on a very tailored behavior, that is, consumers’ willingness to buy meat in mobile slaughter units (Hoeksma et al., 2017). A key dimension in the VBN theory is personal norm, which represents the direct driver of behavior. Personal norm is related to the individual’s belief about what is right to do for a positive self-evaluation (Fransson and Biel, 1997) and relies on interiorized values (Thøgersen, 2006). According to Bamberg et al. (Bamberg et al., 2007; Bamberg and Möser, 2007), personal norm is developed on the basis of social norms, since the latter delivers the standards of behavior that a social reference group applies and views as appropriate in a specific context, that is, what the group considers right or wrong. This was previously found in the context of meat consumption, showing that people form the intention to do something about their beef consumption if they feel a moral obligation to act, which in turn is developed by the expectations from others (Klöckner, 2017).

Social norms include injunctive norms, which concern the perception of what most people approve or disapprove about a person’s behavior (i.e., perception of what other people think one should do) in a given context or situation, and descriptive norms, which refer to the perception related to what the majority of people actually do in that context or situation (Cialdini et al., 1991; Schultz et al., 2008). Both kinds of social norms have proven to influence a variety of pro-environmental behaviors, such as energy saving (Schultz et al., 2007; Ferguson et al., 2011), recycling (Carrus et al., 2009; Fornara et al., 2011), littering (Kallgren et al., 2000), water conservation (Lede et al., 2019), hotel guests’ reuse of towels (Schultz et al., 2008), and adoption of photovoltaic systems (Jager, 2006). Recently, Cheah et al. (2020) found injunctive norms to be positively related to consumers’ attitude and intention to reduce meat consumption.

### The Measurement Issue

One important issue that we address in this paper concerns how to measure actual meat consumption. Most studies on this specific pro-environmental behavior have relied on measures of self-reported behavior (i.e., concerning the past) or behavioral intention (i.e., concerning the future), often represented by a single item. Examples are “How many days per week do you eat meat with your main meal (including chicken)?” (de Boer et al., 2013), “How many days per week do you eat meat (excluding fish)?” (Graham and Abrahamse, 2017), and “How many servings of red meat and processed meat have you eaten in the previous week?” (Carfora et al., 2019). In their systematic review of experimental studies on meat consumption, Harguess et al. (2020) found that only less than 1% of studies measured meat consumption reduction through observable meat avoidance, whereas all the others relied on self-report measures of behaviors or, mostly (i.e., about 67%), intentions/willingness/desire.

In a recent study, van der Werf et al. (2020) have underlined the vulnerability of self-reported data by showing that the self-reported behavior was weakly to fairly correlated to actual food waste behavior. Whybrow et al. (2016) argue that people are inconsistent self-reporters of food intake, which may mislead to wrong conclusions. The limitations of this kind of self-report measures have been commonly acknowledged. A recent review by Lange and Dewitte (2019) pointed out that (i) it is unlikely that all respondents have the same idea of concepts such as “paper,” “recycling,” and “often” (see also Kormos and Gifford, 2014); (ii) typically an item does not ask for a simple behavioral report, but rather for an extensive retrospective survey, which could be affected by memory biases or computing difficulties; (iii) respondents search for consistency across their responses in the questionnaire; and (iv) respondents are prone to compliance with the expectations or preferences of the researcher/interviewer as well as to social desirability pressure.

Therefore, the present paper aimed at both evaluating the effectiveness of a simulated behavior measurement and, as mentioned earlier, at contributing to the literature on the socio-psychological variables involved in meat consumption. Specifically, Study 1 investigated whether the VBN theory – plus social norms – could explain meat consumption. Study 2 investigated whether the choice of purchasing meat could be explained also by a selfish driver such as individuals’ health concern.

## Study 1

### Objective and Hypotheses

The goal of Study 1 was 2-fold.

**Figure 1:**
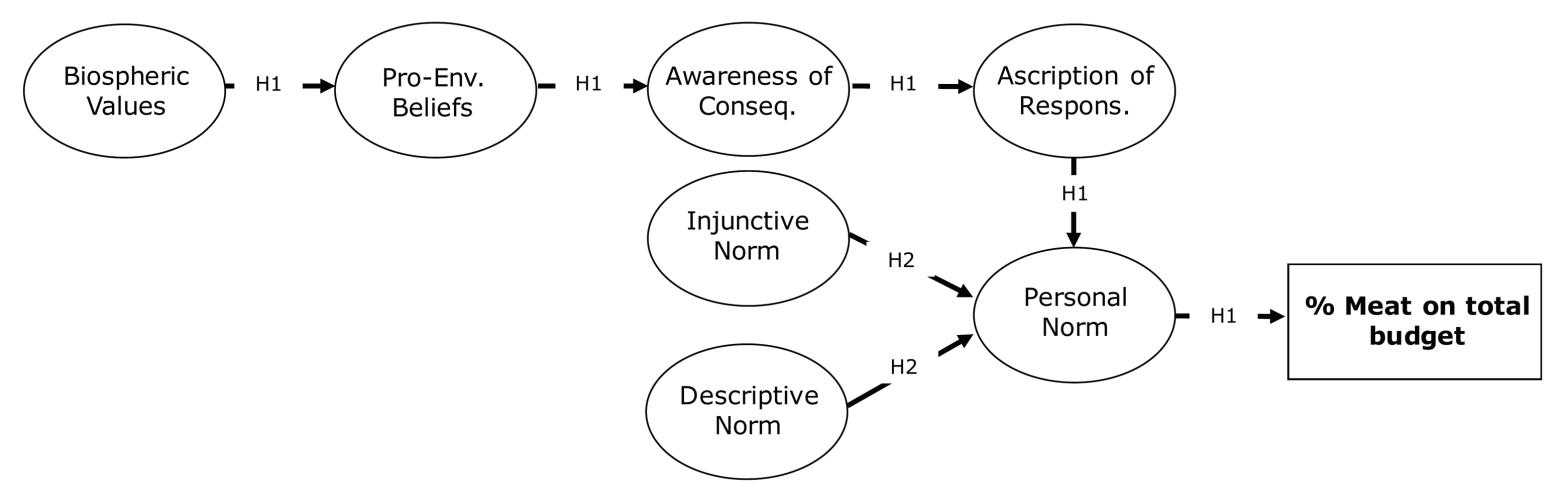
Conceptual model and hypothesized paths of Study 1.

First of all, we wished to test the predictive power of an extended version of the VBN theory (Stern et al., 1999), including social norms, predicting meat purchase (see Figure 1). Specifically, the aim was to verify the importance of the VBN constructs in determining sustainable food choices, taking into consideration the effect of social norms, which have shown to be significant antecedents of personal norm (Bamberg et al., 2007; Bamberg and Möser, 2007; Fornara et al., 2016).

Secondly, we aimed to provide the first validation of a behavioral measure based on a simulation of purchase behavior, which is supposed to resemble actual behavior better than self-report behavioral intention as will be reported later on.

The following research hypotheses were then developed.

*Hypothesis* 1: In line with the VBN theory, meat consumption is expected to be predicted by a sequential chain, which includes, from the most distant to the most proximal, biospheric values, pro-environmental beliefs, awareness of consequences, ascription of responsibility to oneself and, finally, personal norm, which is the closest proxy to the simulated behavior.
*Hypothesis* 2: Personal norm is expected to be predicted by both injunctive and descriptive norms, in line with previous findings (Bamberg et al., 2007; Bamberg and Möser, 2007; Fornara et al., 2016).
*Hypothesis* 3: Meat consumption, i.e. the behavioral simulation measure of food purchase, is expected to show a medium-size correlation^1^ with self-report behavioral intention, thus providing a convergent validity proof.

### Materials and Methods

#### Sample and Procedure

The sample included 198 Italian participants (57.6% females and 41.4% males)^2^, aged between 15 and 74 years (*M* = 31.61 and *SD* = 9.68). In terms of education, the majority of the participants are high school graduates (43.4%), followed by those with a BA degree (27.8%) and a MA degree (15.7%). In lower percentage, we find those who have a doctoral degree or equivalent level of qualification (4%), middle school (8.6%), and primary school (0.5%).

**Figure 2:**
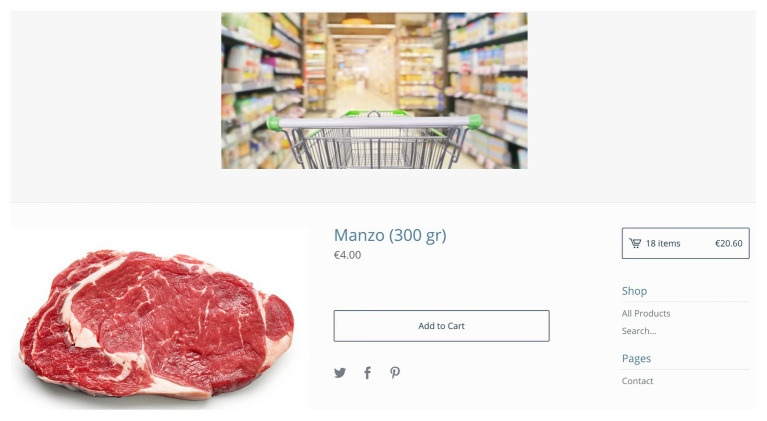
Screenshot of the virtual supermarket. For example, one-item of beef (“Manzo”) corresponded to 300 g. Participants could add to cart the products they wished to buy as many times they wanted within the money range they were given.

Participants who agreed to take part in the study delivered their informed consent and were then invited to the online questionnaire platform, where they read that they would be participating in a study about eating behavior lasting about 15 min. The survey consisted of two parts: in the first part, the participants were redirected to an online supermarket web page (see Figure 2), where they were asked to purchase some food products, as if they would do in reality for their own personal need (not including family members or other members of the household), within a budget set by the experimenters. After the food purchase, their behavioral intention was assessed and in the second part, before filling in the questionnaire, the participants were asked to complete an irrelevant filler task in order to prevent covert rehearsal. Subsequently, the participants were surveyed on the measures of the socio-psychological dimensions of the VBN constructs (values, general pro-environmental beliefs, awareness of consequences, ascription of responsibility, and personal norm), as well as the social norms (injunctive and descriptive), which are detailed in the next section. Therefore, in order to avoid the confounding possibility of the question order effects, such as instances of strategic self-presentation in terms of consistency between attitudes, beliefs, and the subsequent behavior, we decided to assess the simulated behavior (and the behavioral intention) at the first stance.^3^ Finally, the questionnaire included some socio-demographic indicators. Data were collected during December 2017.

#### Measures

The questionnaire included the following measures.

##### Percentage of Meat Products on Total Expenditure

This variable was created by computing the percentage of money (euros) spent for purchasing meat products out of the total amount of expenditure. The choice to measure individuals’ simulated purchase behavior is supported by the fact that it may be considered a direct proxy of food consumption behavior. Therefore, the participants were asked to virtually shop in a supermarket, which included a wide variety of products, from dairy to fruit, vegetables, and cereal based food. The supermarket offered meat-based products as well as other protein-rich alternatives to meat, such as tofu, wheat protein, and vegan cold cut options covering a broad variety of needs. The participants’ virtual experience involved inserting the chosen items in the shopping cart from a minimum amount of 20 euros to a maximum amount of 25 euros. Some examples of the products are the following: “Broccoli (500gr) – €1.10,” “Rice (500gr) – €0.80,” “Sausages (300gr) – €2.20,” “Spaghetti (500gr) – €0.80,” and “Turkey (300gr) – €2.00.” Each product was given a weight and a price based on the average price and package weight of four different supermarkets widely known in the Italian market (Nonna Isa, Despar, Conad, and Crai). All foods were presented on a white background and unpackaged.

##### Behavioral Intention

Individuals’ behavioral intention was measured by a single item: “Think about what you will eat in the next week. How many times do you think you will eat meat or cold cuts?” Participants were asked to answer on a 5-point Likert scale from 0 = *never* to 4 = *twice a day*.

##### Biospheric Values

This variable was measured by using two items (*α* = 0.72) from a shorter version of the 12-item scale of values developed by De Groot and Steg (2008) based on the tripartition proposed by Stern et al. (1998). The items were “Unity with nature: fitting into nature” and “Protecting the environment: preserving nature.” Participants indicated the importance level of each value item as a guiding principle in their lives on a 7-point Likert-type scale from 0 = *the least important* to 6 = *the most important*.

##### General Pro-environmental Beliefs

It was measured through an adaptation of the NHIP (New Human Interdependence Paradigm) scale developed by Corral-Verdugo et al. (2008) and included five items (*α* = 0.93; e.g., “Human beings can enjoy nature only if they make wise use of its resources”). The response scale was a 7-point Likert-type from 0 = *absolutely disagree* to 6 = *totally agree*.

##### Ascription of Responsibility

It was measured through a single item: “The choice of reducing my consumption of meat or cured meats does not depend on me.” Participants were asked to indicate their degree of agreement or disagreement through a 7-point Likert-type scale from 0 = *absolutely disagree* to 6 = *totally agree*.

##### Awareness of Consequences of Meat Consumption

Individual’s awareness was measured by a three-item scale (α = 0.82) adapted from Abrahamse and Steg (2009); e.g., “Eating meat or cured meats every day (or almost) is a risk for the environment.” The response scale was a 7-point Likert-type from 0 = *absolutely disagree* to 6 = *totally agree*.

##### Personal Norm

Individuals’ moral obligation to reduce meat consumption was measured through a three-item scale (*α* = 0.86) adapted from Abrahamse and Steg (2009); e.g., “I feel guilty if I eat meat every day (or almost).” The response scale was a 7-point Likert-type from 0 = *absolutely disagree* to 6 = *totally agree*.

##### Social Norms (Injunctive Norm and Descriptive Norm)

They were measured through three-item scales (injunctive norm *α* = 0.88 and descriptive norm *α* = 0.87) adapted from Fornara et al. (2011). An example of *injunctive norm* items is “Most of my friends would approve my choice to decrease my meat consumption,” while an example of *descriptive norm* items is “Many of my friends are decreasing their consumption of meat.” The response scale was a 7-point Likert-type from 0 = *absolutely disagree* to 6 = *totally agree*.

#### Data Analysis

There was no missing data, since the questionnaire would not proceed to the next question until an answer was provided.

Preliminary analyses (i.e., descriptive statistics and reliability tests and Pearson’s *r* bivariate correlations) were carried out for all variables and scales. A confirmatory factor analysis with the six considered factors was then performed in order to test the measurement model. Mardia’s tests of skewness and kurtosis (Mardia, 1970) were performed to assess multivariate normality. Maximum likelihood estimator with robust standard errors was employed (Yuan and Bentler, 2000). Finally, structural equation modeling (SEM) was run in R version 3.6.1 by using the Lavaan package (Rosseel, 2012) for testing the hypotheses (maximum-likelihood robust estimation method; Bollen, 1989). Stepwise model revisions were undertaken to improve goodness of fit. To assess the overall fit of the model, the chi-square/df ratio (<2.0), the Bentler (1990) Comparative Fit Index (CFI; > 0.90), the Root Mean Square Error of Approximation (RMSEA; < 0.05; Steiger, 1990), and the Standardized Root Mean Square Residual (SRMR; Bentler, 1995) were considered (Hooper et al., 2008).

### Results

Table 1 reports means, standard deviations, and Pearson’s *r* bivariate correlations between the variables^4^ inserted in the SEM analysis. A medium-size correlation emerged between simulated behavior (% of money spent for meat products) and behavioral intention (*r* = 0.41; *p* < 0.001) as expected (H3).

**Table 1:** Descriptive statistics and correlation coefficients of Study 1.

Variable	*M*	*SD*	1	2	3	4	5	6	7
1. Biospheric values	5.07	1.06	1						
2. General pro-env` beliefs	4.85	1.33	0.355^***^	1					
3. Awareness of consequences	3.61	1.33	0.188^**^	0.343^***^	1				
4. Personal norm	2.29	1.87	0.244^**^	0.258^***^	0.616^***^	1			
5. Descriptive norm	1.68	1.44	0.078	0.143^*^	0.398^***^	0.469^***^	1		
6. Injunctive norm	2.13	1.56	0.076	0.159^*^	0.336^***^	0.438^***^	0.497^***^	1	
7. Behavioral intention	1.77	0.83	−0.275^***^	−0.212^**^	−0.272^***^	−0.346^***^	−0.268^***^	−0.189^**^	1
8. % of money spent for meat	24.27	15.09	−0.231^**^	−0.119	−0.215^**^	−0.357^***^	−0.189^**^	−0.039	0.407^***^

* *p* < 0.05;

** *p* < 0.01;

*** *p* < 0.001.

The Mardia’s tests of skewness and kurtosis (Mardia, 1970) indicated the deviation from multivariate normality (_1,p_ = 84.99, *p* < 0.001; _2,p_ = 477.062, *p* < 0.001). Therefore, a maximum likelihood estimator with robust standard errors was employed (Yuan and Bentler, 2000). The confirmatory factor analysis showed evidence of good fit (*χ*^2^_137_ = 201.155, *p* < 0.001; *χ*^2^/df = 1.533; CFI = 0.971; RMSEA = 0.050; SRMR = 0.052) with standardized factor loadings further confirming the distinctive variables (see Table 2).

**Table 2:** Parameter estimates from the six-factor CFA of Study 1.

Latent factor	Indicator	*B*	*SE*	*Z*	*β*	Sig.
Biospheric values	bio1	1.000	0.000		0.763	
	bio2	1.403	0.239	5.876	0.774	***
General pro-env` beliefs	nhip1	1.000	0.000		0.898	
	nhip2	0.889	0.047	19.056	0.825	***
	nhip3	0.921	0.047	19.702	0.893	***
	nhip4	0.914	0.060	15.353	0.846	***
	nhip5	0.789	0.072	10.890	0.842	***
Awareness of consequences	ac1	1.000	0.000		0.862	
	ac2	0.859	0.087	9.913	0.694	***
	ac3	0.978	0.105	9.328	0.769	***
Personal norm	pn1	1.000	0.000		0.796	
	pn2	1.003	0.076	13.267	0.849	***
	pn3	0.969	0.070	13.814	0.818	***
Descriptive norm	des1	1.000	0.000		0.755	
	des2	1.075	0.115	9.338	0.865	***
	des3	1.104	0.127	8.678	0.870	***
Injunctive norm	inj1	1.000	0.000		0.808	
	inj2	0.941	0.075	12.576	0.889	***
	inj3	0.991	0.066	15.082	0.872	***

The factor loading of the first indicator of each latent variable is fixed to 1. *SE* = standard error; *B* = non-standardized estimate; *β* = standardized estimate; Sig. = values of *p* corresponding to the z-statistic. ^*^p < 0.05; ^**^p < 0.01; ^***^p < 0.001.

**Figure 3:**
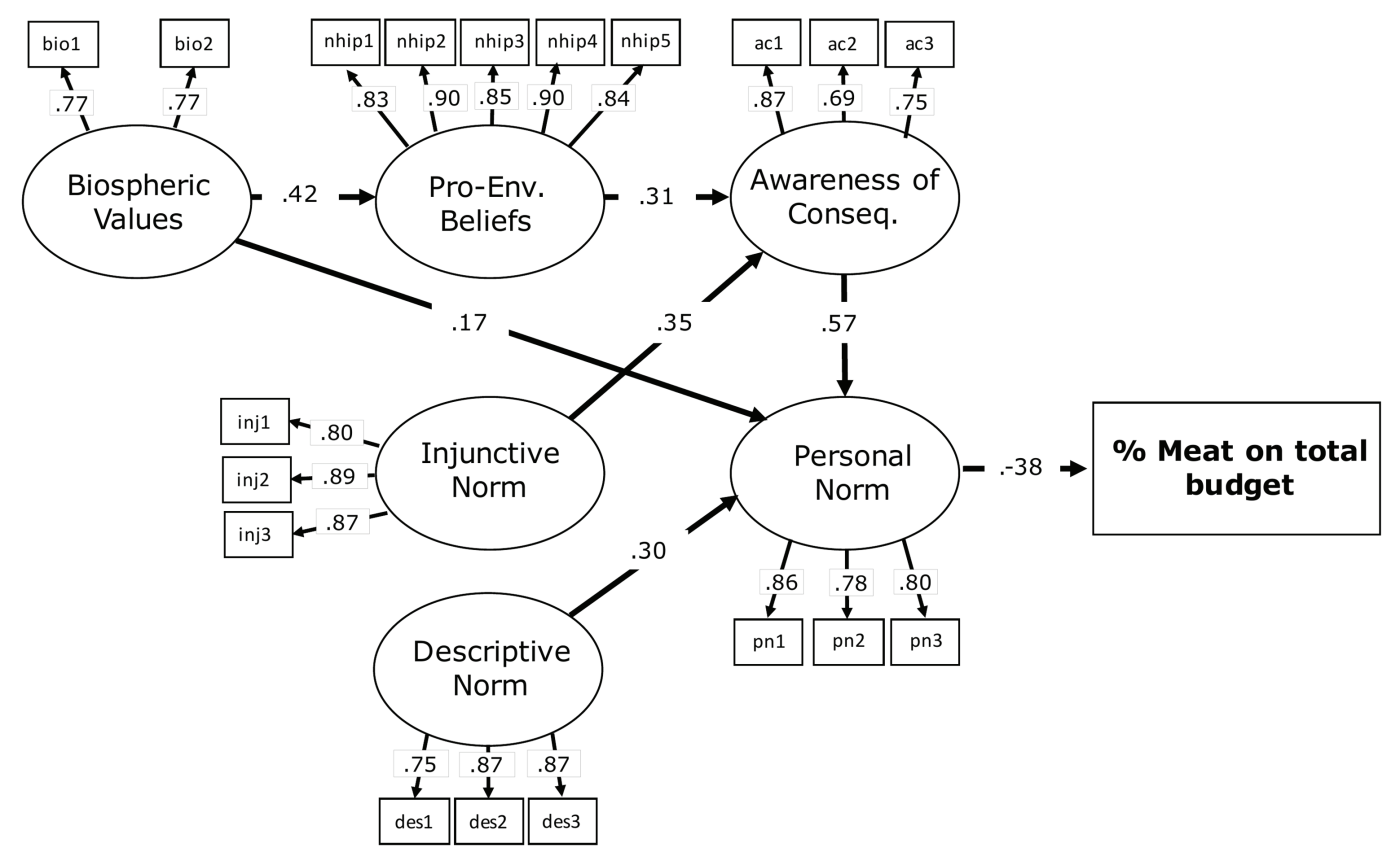
Standardized direct effects and item loadings in the structural model of Study 1. To simplify presentation, the variances – curved double arrows – of the exogenous latent variables and the standardized error variances are not reported.

The structural model showed a good fit to the data: *χ*^2^_161_ 236.682, *p* < 0.001; *χ*^2^/df = 1.548; CFI = 0.960; RMSEA = 0.050; and SRMR = 0.075. Standardized coefficients of the path model are shown in Figure 3.

Concerning H1 and H2, the outcome variable percentage of purchased meat was negatively predicted by personal norm (*β* = −0.38; *p* < 0.001), in turn, personal norm was positively predicted by awareness of consequences (*β* = 0.57; *p* < 0.001), biospheric values (*β* = 0.17; *p* < 0.05), and by descriptive norm (*β* = 0.30; *p* < 0.001), followed by awareness of consequences, which was positively predicted by pro-environmental beliefs (*β* = 0.31; *p* = 0.001) and by injunctive norm (*β* = 0.35; *p* < 0.001), successively, pro-environmental beliefs was positively predicted by biospheric values (*β* = 0.42; *p* < 0.001). The expected direct paths within the VBN were confirmed apart from the relationship between ascription of responsibility and, respectively, personal norm and awareness of consequences. Therefore, ascription of responsibility was excluded from the model; nevertheless, as mentioned above, awareness of consequences predicted significantly personal norm.

### Discussion

Study 1 provided the first proof of convergent validity regarding the proposed new measure, since the simulated behavior showed a significant medium-size correlation with behavioral intention, thus confirming H3.

Overall, our results support the extended model of the VBN (Stern et al., 1999) including social norms. Specifically, the expected VBN sequence of unidirectional paths among the variables taken into consideration, i.e., respectively, values, general pro-environmental beliefs, awareness of consequences, personal norm, and (simulated) behavior emerged.

Consistently with the VBN theory and previous findings, biospheric values were positively correlated to both pro-environmental beliefs and personal norm (Stern et al., 1999; Hunecke et al., 2001; Nordlund and Garvill, 2002; Steg et al., 2005; Fornara et al., 2016). In other words, the stronger an individual’s pro-environmental values are the more they will feel an obligation for protecting the environment and, therefore, for reducing their consumption of meat. As regards the role of both injunctive and descriptive social norms as direct antecedents of personal norm, consistently with H2, the prediction of descriptive norm was confirmed, in line with the conception of social norms as internalized standards that provide the content of an individual’s moral standards (Bamberg et al., 2007; Bamberg and Möser, 2007). Consequently, the belief that people surrounding the individual are reducing their meat consumption should activate her/his moral obligation to follow in the same direction. On the other hand, the connection between injunctive norm and personal norm was indirect, *via* awareness of consequences, thus indicating, in line with previous research (see Fornara et al., 2016), that the stronger the perception that significant others approve one’s own reduction of meat consumption, the higher the individual’s awareness about the consequences of such behavior will be. Furthermore, people care whether their behavior is moral to others and are motivated to maintain a positive moral self-image (Jordan and Monin, 2008) and to belong to a moral group (Ellemers and van den Bos, 2012).

Study 1 thus confirmed the link between biospheric values and pro-environmental behaviors, showing that individuals with predominant biospheric values act evaluating costs and benefits of their behaviors for the environment. However, an important question remains unanswered, regarding those individuals who tend to consider the benefits and the costs for themselves. Research has shown that egoistic values are, generally, negatively related to pro-environmental behaviors (De Groot and Steg, 2008; Stern and Dietz, 1994; Stern et al., 1998), but what happens when the egoistic, utilitarian pattern is represented by concern for one’s own health? As underlined in the introduction, food consumption is a target pro-environmental behavior, which could be also positively oriented by a selfish driver reflecting individual (cost/benefit) considerations like health concern (Bertolotti et al., 2016, 2020; Jagers et al., 2017; Carfora et al., 2019; Sanchez-Sabate and Sabaté, 2019), indicating that meat consumption is a behavior influenced by both individual and environmental considerations. This highlights the importance of using a comprehensive set of psychological variables in relation to this specific behavior. In order to shed light on this point, we designed the following study.

## Study 2

### Objectives and Hypotheses

**Figure 4:**
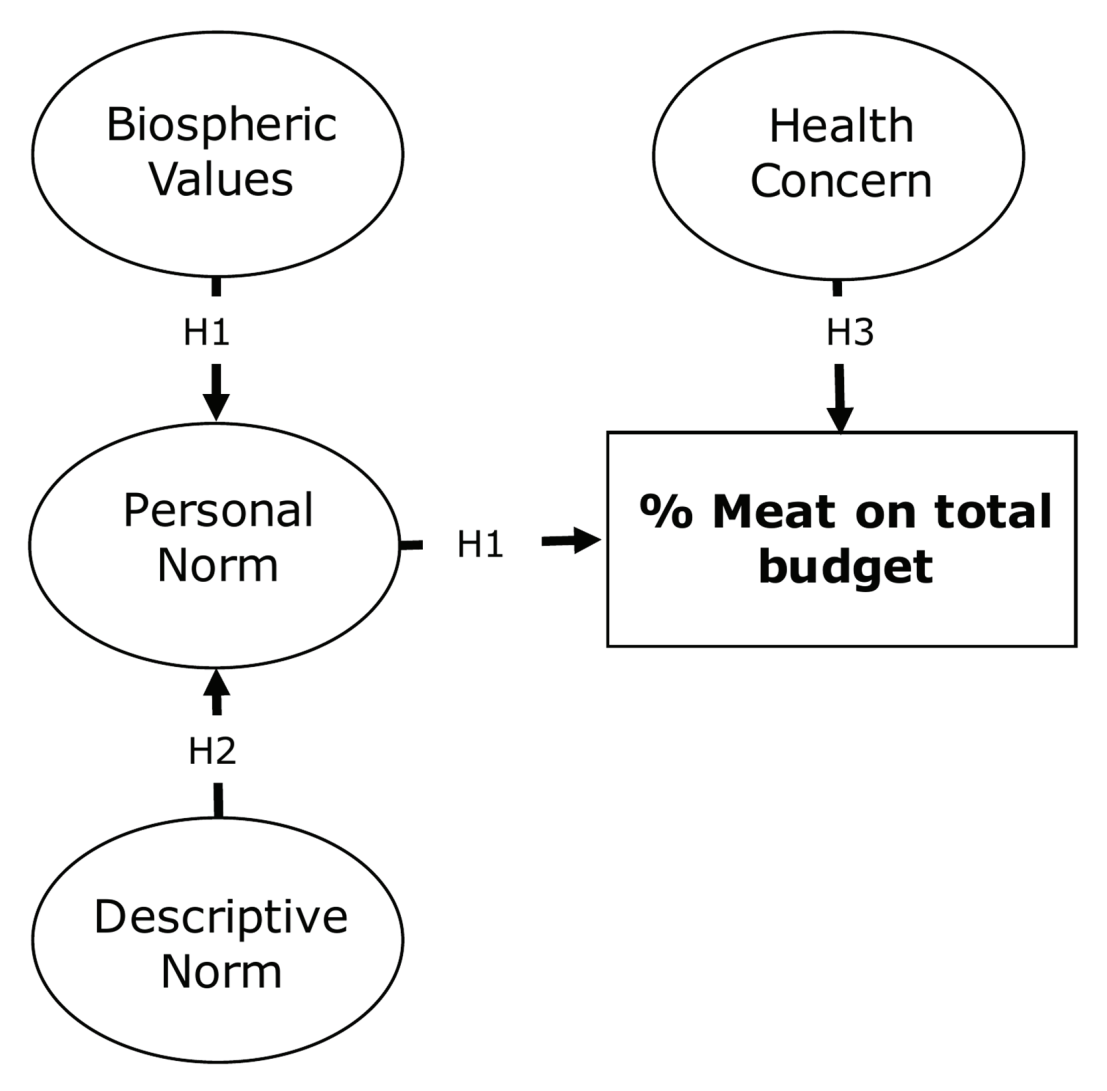
Conceptual model and hypothesized paths of Study 2.

The main goal of Study 2 was to verify whether the amount of meat purchase could also be motivated by a healthy food concern, that is, the importance of eating healthily for one’s own health (Tudoran et al., 2009). Therefore, in addition to the main predictors included in Study 1 (i.e., biospheric values, personal norm, and descriptive norm), a measure of health concern was included and tested in a more parsimonious model (see Figure 4).

Secondly, Study 2 aimed at providing a further convergent validation of the virtual shopping measure, by using a comparative measure of behavioral intention more robust than the single item of Study 1.

Hence, we formulated the following hypotheses.

*Hypothesis* 1: Consistently with the VBN framework and Study 1, biospheric values are antecedents of personal norm, which in turn predicts lower meat purchase.
*Hypothesis* 2: Descriptive norm predicts personal norm, confirming Study 1.
*Hypothesis* 3: Healthy food concern predicts lower meat purchase.
*Hypothesis* 4: Meat purchased has a medium-size correlation with a behavioral intention scale, thus providing a further proof of convergent validity of the proposed new measure.

### Materials and Methods

#### Sample and Procedure

The sample consisted of 218 Italian participants (64.8% females and 34.8% males), aged between 18 and 54 years (*M* = 26.7; *SD* = 7.0). The majority of the participants are high school graduates (44.4%), followed by those with a BA degree (30%) and a MA degree (17.6%). In lower percentage, we find those who have a doctoral degree or equivalent level of qualification (6.8%), and middle school (1.2%). Participants were surveyed with the same procedure of Study 1, therefore, the survey was completed in the same order (purchasing behavior, intention, socio-psychological dimensions, and socio-demographic indicators). Data were collected during April 2018.

#### Measures

The online questionnaire used for this study included some measures, which were identical to the ones used for Study 1, that is biospheric values (*α* = 0.73), personal norm (*α* = 0.83), percentage of meat purchased on total expenditure, and the socio-demographics. Moreover, the following measures were added or improved from Study 1.

##### Behavioral Intention

In order to rely on a measure more reliable than the one used for Study 1, this variable was measured by a five item scale (*α* = 0.81) including items such as “How many times do you think you will buy meat next week?” Participants were asked to answer the question using a 5-point Likert scale from 0 = *never* to 4 = *twice a day*.

##### Health Concern

This variable was measured by using the two items (*α* = 0.67)^5^ referring to food health concern from Tudoran et al. (2009) health value scale; i.e., “I think of myself as a person who is concerned about healthy food” and “I’m very concerned about the health-related consequences of what I eat.” The response scale was a 7-point Likert-type from 0 = *absolutely disagree* to 6 = *totally agree*.

#### Data Analysis

As for Study 1, descriptive and reliability analyses were conducted for all variables and scales, and Pearson’s r bivariate correlation was run between simulated meat consumption and the aggregate measure of behavioral intention. Levels of skewness and kurtosis were based on Mardia’s test (Mardia, 1970). A confirmatory factor analysis was performed to test the measurement model and the robust version of the maximum likelihood estimator was chosen (Yuan and Bentler, 2000). Finally, the structural model was tested with the assumed paths. The overall fit of the model was assessed by using the same criteria of Study 1. Both CFA and SEM were performed using the Lavaan package (Rosseel, 2012) within the R environment (version 3.6.1).

### Results

Means, standard deviations and Pearson bivariate correlations of all variables are presented in Table 3.

**Table 3:** Descriptive statistics and correlation coefficients of Study 2.

Variable	*M*	*SD*	1	2	3	4	5
1. Biospheric values	5.05	0.98	1				
2. Personal norm	2.86	1.84	0.254^***^	1			
3. Descriptive norm	1.92	1.48	0.139^*^	0.525^***^	1		
4. Health concern	3.89	1.26	0.306^***^	0.348^***^	0.214^**^	1	
5. Behavioral intention	2.27	0.61	−0.132	−0.494^*^	−0.263^***^	−0.494^***^	1
6. % of money spent for meat	26.37	15.19	−0.135^*^	−0.350^***^	−0.169^*^	−0.350^***^	0.536^***^

* *p* < 0.05;

** *p* < 0.01;

*** *p* < 0.001.

The Mardia’s tests of skewness and kurtosis (Mardia, 1970) indicated deviation from multivariate normality (_1,p_ = 14.763, *p* < 0.001; _2,p_ = 139.085, *p* < 0.001). Therefore, a maximum likelihood estimator with robust standard errors was employed (Yuan and Bentler, 2000). The confirmatory factor analysis showed evidence of good fit (*χ*^2^_29_ = 36.607, *p* > 0.05; *χ*^2^/df = 1.301; CFI = 0.989; RMSEA = 0.035; SRMR = 0.041) with standardized factor loadings further confirming reliable variables (see Table 4).

**Table 4:** Parameter estimates from the four-factor CFA of study 2.

Latent factor	Indicator	*B*	*SE*	*Z*	*β*	Sig.
Biospheric values	bio1	1.000	0.000		0.822	
	bio2	0.998	0.273	3.653	0.702	***
Personal norm	pn1	1.000	0.000		0.779	
	pn2	1.088	0.083	13.122	0.836	***
	pn3	0.883	0.087	10.168	0.752	***
Descriptive norm	des1	1.000	0.000		0.732	
	des2	1.104	0.087	12.735	0.892	***
	des3	1.122	0.095	11.748	0.903	***
Health concern	hc1	1.000	0.000		0.523	
	hc2	2.436	0.412	5.919	0.765	***

The factor loading of the first indicator of each latent variable is fixed to 1. *SE* = standard error; *B* = non-standardized estimate; *β* = standardized estimate; Sig. = values of *p* corresponding to the z-statistic. ^*^p < 0.05; ^**^p < 0.01; ^***^p < 0.001.

**Figure 5:**
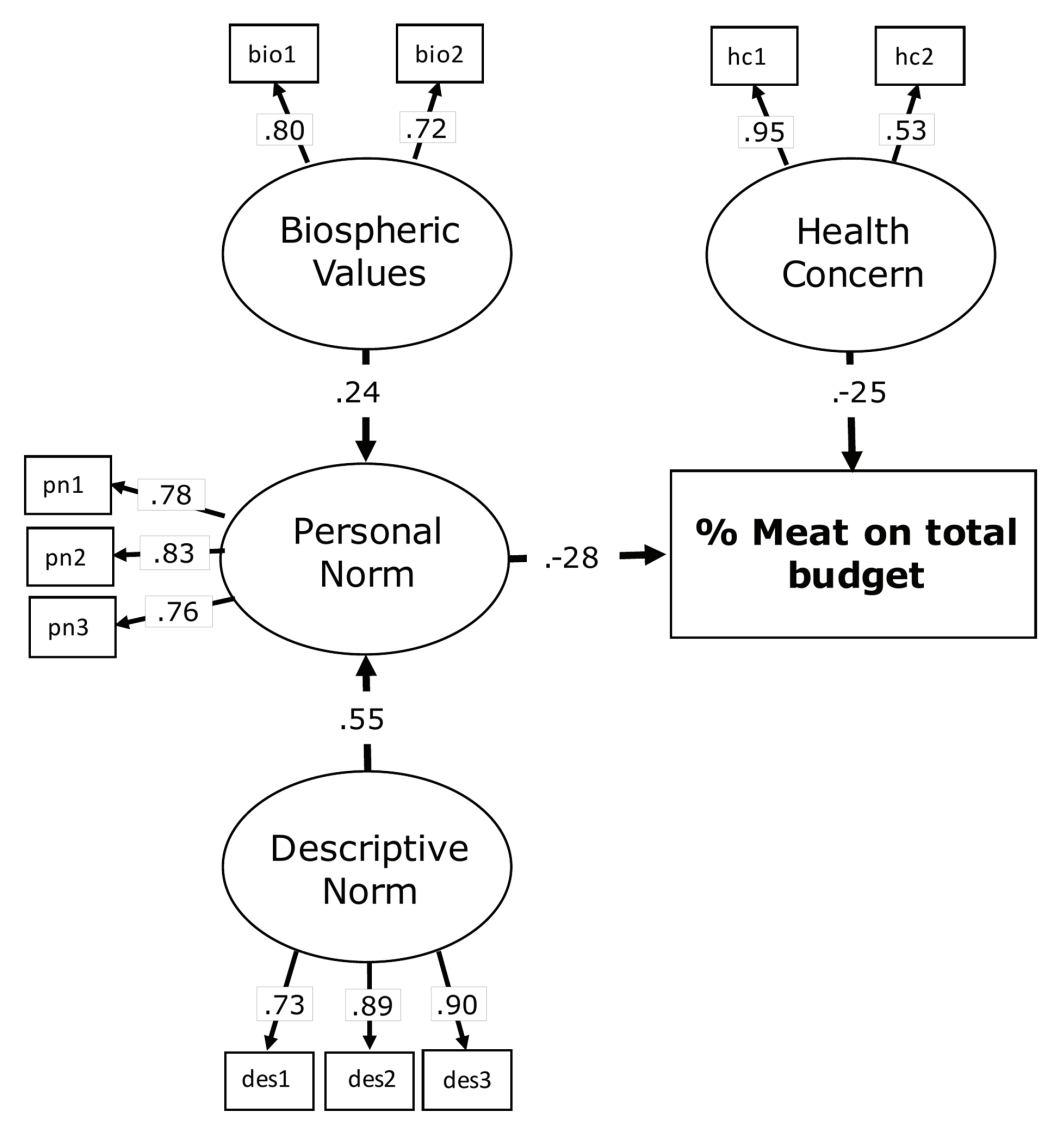
Standardized direct effects and item loadings in the structural model of Study 2. To simplify presentation, the variances – curved double arrows – of the exogenous latent variables and the standardized error variances are not reported.

Figure 5 reports the tested model, including the standardized coefficients of the links. The model presented a good overall fit to the data: *χ*^2^_55_ 67.31, *p* < 0.01; *χ*^2^/df = 1.81; CFI = 0.968; RMSEA = 0.060; and SRMR = 0.058.

In line with H1, biospheric values predict the personal norm (*β* = 0.24; *p* < 0.05), which in turn has a direct negative association with percentage of meat purchased (*β* = −0.28; *p* < 0.001). Concerning H2, descriptive norm is significantly related to personal norm (*β* = 0.55; *p* < 0.001) and, consistently with H3, healthy food concern is negatively linked to the simulated behavior (*β* = −0.25; *p* < 0.001).

As for H4, the Pearson’s bivariate correlation indicate a relevant association between simulated behavior (% of money spent on meat) and behavioral intention (*r* = 0.54; *p* < 0.001).

### Discussion

Study 2 provided a further convergent validation of the virtual shopping measure. In fact, the correlation between percentage of meat products purchased and the scale measuring behavioral intention (i.e., a more reliable than the single item used in Study 1) was even higher than the one found in Study 1, but again its size was not too large, thus confirming that these measures tap different patterns – though related – i.e., respectively, intention and (simulated) behavior.

Besides the confirmation of the “pro-environmental” path stemming from biospheric values and personal norm, Study 2 evidenced the distinct influence of a selfish driver reflecting individual considerations, such as food health concern, in orienting meat consumption patterns. Specifically, SEM results confirm H1, by showing a pathway in which biospheric values are antecedents of personal norm, which in turn predicts meat consumption reduction. This is consistent with Study 1 and the VBN theory (Stern et al., 1999). In line with H2, the link between descriptive social norm and personal norm is also confirmed, consistently with Study 1 and previous research that conceptualizes both social norms as internalized standards that provide the content of an individual’s personal norms (Bamberg et al., 2007; Bamberg and Mӧser, 2007). Finally, healthy food concern negatively predicts the percentage of meat consumption in the virtual supermarket confirming H3 in line with previous findings (Mullee et al., 2017; Malek et al., 2019; Sanchez-Sabate and Sabaté, 2019; Bertolotti et al., 2020).

## General Discussion

Overall, the results substantially support the direct role played by individuals’ sense of moral obligation to reduce meat consumption, the indirect role of individuals’ value system, and the influence of significant others on the simulated purchasing behavior together with individual’s health concern.

In detail, the first study confirmed the role of the constructs of the VBN model in predicting the overall meat purchase, and this is consistent with the literature regarding other pro-environmental behaviors (e.g., Stern et al., 1999; Nordlund and Garvill, 2003; Kaiser et al., 2005; Steg et al., 2005; Ibtissem, 2010; Jansson et al., 2011; Fornara et al., 2016; Han et al., 2017). The lack of the hypothesized direct links concerning ascription of responsibility (i.e., as antecedent of personal norm and predicted by awareness of consequences) is partially compensated by the link between awareness of consequences and personal norm, which thus mirrors a one-level jump within the VBN sequential chain. This was also found in previous research explaining other environmental behaviors, such as consumer behavior, environmental citizenship, willingness to sacrifice, willingness to reduce car use (Stern et al., 1999; Nordlund and Garvill, 2003), and household renewable energy use (Fornara et al., 2016).

Consistently with the VBN theory, biospheric values were positively correlated to both pro-environmental beliefs and personal norm (Stern et al., 1998, 1999; Hunecke et al., 2001; Nordlund and Garvill, 2002; Steg et al., 2005, 2011; Steg and De Groot, 2012; Fornara et al., 2016), indicating that those who highly value the quality of the environment feel more obliged to reduce their meat consumption. This is in line with previous research indicating a positive association between self-transcendent values and self-determined motivations to act pro-environmentally (De Groot and Steg, 2010).

Furthermore, Study 1 confirmed the role of social norms, indicating that the belief that significant others are reducing their meat consumption (i.e., descriptive norm) may activate individuals’ moral obligation to follow the same direction, which is consistent with the concept that social norms provide the internalized content of an individual’s moral standard (Bamberg et al., 2007; Bamberg and Mӧser, 2007). At the same time, the extent to which significant others approve the choice to limit one’s own meat consumption (i.e., injunctive norm) indirectly impacts individuals’ moral obligation to reduce meat consumption by informing about the environmental consequences of meat consumption. The lack of the direct effect of the injunctive norm on personal norm might be related to a form of psychological reactance, being one’s autonomy threatened by the perceived social pressure.^6^ Fornara et al. (2016) found the same effect of the injunctive norm on awareness of consequences in the context of adopting renewable energy sources at the household level, supporting our finding (i.e., what others approve may still be important in informing the awareness of the environmental consequences). However, we find both social norms to be quite low on average, indicating that people perceive a moderate pressure to reduce their meat consumption. Therefore, further studies should provide additional understanding with regard to this relationship. Nevertheless, these results together should bring our attention to the importance of considering possible different sequential paths depending on the specific context of the pro-environmental behavior.

The second study comprised a short version of the VBN “pro-environmental” path leading from biospheric values (and descriptive norm) to meat consumption through the feeling of obligation to act (personal norm), with the addition of a “pro-self” health-related path – which leads from health concern to individuals’ meat purchase. This is in line with studies showing that health motives are among the main motivators in reducing or quitting meat consumption (Mullee et al., 2017; Malek et al., 2019; Sanchez-Sabate and Sabaté, 2019; Bertolotti et al., 2020). Our findings are consistent with previous research highlighting the importance of both environmental and health beliefs in relation to meat consumption (de Boer et al., 2017; Jagers et al., 2017). In essence, as Hofmann et al. (2018, p. 2) pointed out, “[…] resisting the desire to enjoy a steak to help promote a sustainable planet is an instance of moral self-control; resisting the very same desire in order to improve one’s own health is an instance of non-moral self-control.” However, framing a behavior as a moral issue has the advantage to last in the long-term (Steg, 2016), to evoke positive affect when adopted (Bolderdijk et al., 2013), to provide a warm glow effect (Taufik, 2018), to be a distinct group-based guideline (Ellemers et al., 2013), and to motivate people to influence others in society (Skitka et al., 2005; Feinberg et al., 2019). This may be the case because framing a behavior as a moral issue might activate individual’s moral self-view (Aquino and Reed, 2002), which is directly related to their own behavior and might make salient the backlash of a negative evaluation in terms of morality (Pagliaro, 2012; Leach et al., 2015; Pagliaro et al., 2016).

Study 2 further confirmed the role of the social descriptive norm on pro-environmental behaviors, showing that individuals’ meat purchase is related to the extent to which people think that significant others are reducing their amount of meat intake. However, Lacroix and Gifford (2019) found social considerations, in terms of social conformity and social influence, to be the lowest reported motivator for those making conscious efforts to reduce meat consumption, while health and environmental aspects were reported among the most important motivators. In a similar way, Nolan et al. (2008) found that participants considered the behavior of their neighbors as the less important impacting their energy conservation despite the fact that the descriptive norms constituted, indeed, the strongest predictors. This was not our case as health concern showed a stronger correlation, on our outcome variable, than the descriptive norm when looking at the direct relations on the correlation matrix. However, the take-home message here is that one of the advantages of using social norms in an applied setting relies on the fact that the general public is not fully aware of the effects of social influence on their own behavior. For this reason, interventions using social influence to target meat consumption may actually produce long-lasting behavioral change due to the fact that individuals’ would perceive their change as intrinsically motivated (Jaeger and Shultz, 2017; see also Barth et al., 2016).

Nevertheless, it is important to mention that we found that, in both studies, the means of the behavioral intention and moral and both social norms were considerably low^7^, while the means of health concern in Study 2 were higher. Furthermore, the mean of biospheric values was the highest, both in Study 1 and Study 2, indicating that the participants on average have high concern for their own health as well as for environmental issues. Nonetheless, with regards to those who intend consuming less meat and have a moral sense of obligation to reduce their meat consumption and are supported by the social environment around them, we find our hypothesis met. Future studies might be directed to further confirm the paths highlighted in the present research.

Finally, a crucial contribution of this research was contingent upon the first proof of validity of a simulated purchasing behavior tool, which provides a more ecological outcome measure to rely on than the traditionally used self-report measurements and, consequently, supports with more methodological strength our findings. In fact, as mentioned in the introduction, measures of self-reported behavior may present issues regarding validity together with limitations regarding specifically pro-environmental behaviors (Whybrow et al., 2016; Lange and Dewitte, 2019; van der Werf et al., 2020). By using a simulated behavior as an outcome measure, the limitations of using a survey to assess individuals’ intentions were overcome.

Regarding future implications, this research provides a contribution to help professionals develop solid strategies, through the management of the cognitive and regulatory processes involved in the production of pro-environmental choices. Indeed, different psychological variables play an important role in the explanation of meat consumption, highlighting the importance of incorporating a comprehensive set of theory-based psychological variables. In this regard, we underline the importance of individuals’ set of values to encourage conscious pro-environmental behaviors. In particular, individuals with self-transcendence values (i.e., biospheric values) are more drawn to develop a sense of moral obligation to act in favor of the environment (Nordlund and Garvill, 2002; Poortinga et al., 2004; Steg et al., 2005); therefore, focusing on personal norms is critical to this purpose as suggested by Harland et al. (1999). At the same time, it is essential to take into account factors reflecting individual (cost/benefit) considerations especially for individuals who do not fall in the self-transcendence domain but are rather (or also) drawn by concerns for their own health.

Reduction in meat consumption for environmental reasons is, nowadays, a minority norm. Framing a message based on trending norms (by portraying a behavior as increasing in popularity) might be one of the most suitable and powerful ways to benefit from social norms with regard to this specific behavior (Mortensen et al., 2019).

Among the limitations of this research, first of all, this is a cross-sectional study, therefore, it is not known whether and how much the consumer behavior will be maintained over time. Moreover, the correlational nature of this study does not allow for conclusions regarding causality about the unidirectional paths tested, providing no evidence of the temporal relationship between predictors and outcome. This issue should be addressed through the development of longitudinal research designs, measuring the predictors first and the dependent variables after a certain period of time (e.g., 1 or 2 weeks) to both avoid order effects and preserve the chronological order of the hypothesized process. However, the fact that these links are theory-based and are coherent with previous research findings focusing on other environmental behaviors provides us with relevant arguments in this direction.

A further limitation concerns the validation of the outcome variable. In fact, the simulation measurement of purchasing behavior was validated with an *ad hoc* self-report measure rather than with a more objective established measure. Thus, even though the use of this procedure for the verification of convergent validity is consistent with previous literature (e.g., Armitage and Conner, 2001; Webb and Sheeran, 2006), future studies are needed to provide a further validation of this measure.

Another issue regards the generalization of the findings in this context of study to other geo-cultural contexts. In fact, we cannot exclude that cultural aspects related to the consumption of meat in the geographical regions to which the sample belongs may have had an influence. It has been shown, in fact, that people’s pro-environmental behavior may vary across different cultural backgrounds (Oreg and Katz-gerro, 2006) and concerns about the environment do not always lead to pro-environmental behavior, because of the influence of other socio-psychological barriers (Tam and Chan, 2017). In order to assess whether these findings can be considered pancultural, we address to future research the duty to verify the reliability of these results in other cultural contexts.

Further analyses and discussion could also be addressed to the role of other types of values for predicting the target behavior. Hedonic values, for example, reflect a focus on individuals’ care about comfort and pleasure. The role of such values for understanding environmentally relevant beliefs, preferences, and actions has been previously acknowledged (Steg et al., 2014). However, enjoying food as an indicator of hedonic values may act as a barrier to reducing meat consumption, which may be an important factor that has not been included in this study. Consistently, the hedonic motivation of perceived tastiness of meat was in fact found to be an important barrier to moralization of meat (Feinberg et al., 2019).

## Conclusion

Meat consumption is a prominent global cause of mortality and environmental degradation. The present study provides support for the predictive validity of the VBN constructs and social norms in explaining meat consumption. Moreover, findings confirmed the important role of healthy eating concern in individuals’ food purchases and gave the first proof of validity of a simulated food purchase measure. The results underline the importance to address individuals’ health and environmental concerns of dietary choices during interventions. As Verain et al. (2016) previously discussed, it is possible to screen consumers on their different cognitive mind-sets and provide them with tailored interventions to promote sustainable food choices. The segmentation framework proposed here regards individuals’ values and concerns. Therefore, regarding the implications for future consumer policy, consumers with a predominant environmental concern may benefit more from environmentally tailored communication while consumers with a predominant health concern may benefit from a communication based on the health consequences of such behavior. In essence, in order to reach a wider population, it would be preferable to develop an approach that combines multiple values regarding food choices, including health and nature-related values, as also suggested by de Boer et al. (2013). Developing an approach that underlines that eating less meat is a choice made by other people because of environmental and health concerns would allow both to target individual’s values and dispositions to conform to social rules.

Interestingly, recent research has shown that foods related to the highest negative environmental impacts are consistently related to the highest increases in disease risk (Clark et al., 2019). This means that actually having a healthier diet would generally improve environmental sustainability.

## Data Availability Statement

The raw data supporting the conclusions of this article will be made available by the authors, without undue reservation.

## Ethics Statement

The studies involving human participants were reviewed and approved by Ethical Committee of the Department of Education, Psychology, Philosophy of the University of Cagliari. Written informed consent from the participants’ legal guardian/next of kin was not required to participate in this study in accordance with the national legislation and the institutional requirements.

## Author Contributions

FF and SP developed the research idea and revised the manuscript. FF, SP, FT, and AL were involved in the planning of both Study 1 and Study 2. FT set-up the online supermarket. AL collected the data. AL and FT performed the data analysis, wrote the manuscript, and contributed equally to this work. All authors contributed to the article and approved the submitted version.

### Conflict of Interest

The authors declare that the research was conducted in the absence of any commercial or financial relationships that could be construed as a potential conflict of interest.

## Footnotes

^1^The hypothesized size of the correlation should not be too large in line with the literature on the overall relationship between intention and behavior (i.e., a medium/large change in intention is supposed to lead to a small/medium change in behaviors, see Webb and Sheeran, 2006).

^2^1% NA.

^3^Our rationale was to prevent participants from being biased in their choices. The process of being influenced by earlier questions is known as “question order – context effect,” which biases the thinking of the respondents and can produce measurement errors (Cassino and Erisen, 2010; Van de Walle and Van Ryzin, 2011). In everyday life, before entering a supermarket or ordering food online, people are not asked to reflect on their own values and to think about environmental and health consequences of their purchases, nor reflect on what significant others do. The adoption of this procedure allowed us to simulate a real-world setting as much as possible, albeit within the limits of a simulated setting. The same procedure was recently being adopted by Gu et al. (2020), which first measured their DV (pro-environmental behavior frequencies) before completing items, which were expected to prime participants’ responses. There is also experimental evidence suggesting that the priming people with a food product (e.g., *via* a social norms’ intervention) leads people to increase the purchase of that product (Richter et al., 2018).

^4^Aggregates were computed for multi-item variables.

^5^Since the variable was composed only by two indicators and Cronbach’s alpha is sensitive to the number of items, it was decided to keep it as a latent variable in spite of consistency scores slightly below 0.70 (factor loadings further confirming internal consistency of the scale can be seen in Table 2).

^6^People’s freedom to eat whatever they please – which is strongly rooted in habits, customs, and traditions ‐ may be undermined by the perception of what others approve, which, in turn, may lead to engage in cognitive dissonance strategies. These strategies might act as a barrier to the moralization of meat consumption (Feinberg et al., 2019). In the same way, de Boer et al. (2013, p. 6) argued that “Contextual factors perceived by consumers as external pressure on their own judgment are expected to lead to negative impacts on their motivation.”

^7^At the same time, we found that (in Study 1) awareness of consequences on average is higher, which is inconsistent with previous findings (see Hartmann and Siegrist, 2017) in which the majority of the studies suggest that consumers are not aware that the production and consumption of meat has a huge impact on the environment. This may be explained by the fact that the environmental consequences of meat consumption might be becoming increasingly popular since the studies reported in the systematic review of Hartmann and Siegrist (2017) date back to 2008–2015.
